# Prednisolone induces apoptosis in corneal epithelial cells through the intrinsic pathway

**DOI:** 10.1038/s41598-017-04509-8

**Published:** 2017-06-23

**Authors:** Jin Suk Ryu, Jung Hwa Ko, Mee Kum Kim, Won Ryang Wee, Joo Youn Oh

**Affiliations:** 1 Laboratory of Ocular Regenerative Medicine and Immunology, Biomedical Research Institute, Seoul National University Hospital, 101 Daehak-ro, Jongno-gu, Seoul, 110-744 Korea; 2 Department of Ophthalmology, Seoul National University Hospital, 101 Daehak-ro, Jongno-gu, Seoul, 110-744 Korea

## Abstract

Glucocorticoid eye drops are one of the most widely used medications in ophthalmology. However, little is known about the effects of glucocorticoids on corneal epithelial cells that are directly exposed to topically-administered glucocorticoids. Here we investigated the effects of prednisolone, a synthetic glucocorticoid analogue frequently used in the clinic, on corneal epithelial cells. Results showed that prednisolone decreased survival of corneal epithelial cells by inhibiting proliferation and inducing apoptosis in a dose-dependent manner. The levels of mitochondrial reactive oxygen species (mtROS), cleaved caspase-3, and -9 were increased by prednisolone. The effects of prednisolone on apoptosis and mtROS were blocked 1) by the glucocorticoid receptor (GR) antagonist RU-38486, 2) in cells with GR siRNA knockdown, and 3) by treatment with N-acetylcysteine. Transcript levels of pro-inflammatory cytokines were increased in corneal epithelial cells upon hyperosmolar stress, but repressed by prednisolone. In NOD.B10.H2^b^ mice, topical administration of 1% prednisolone increased apoptotic cells in the corneal epithelium. Together, data indicate that prednisolone induces apoptosis in corneal epithelial cells through GR and the intrinsic pathway involving mtROS, caspase-9, and -3. The pro-apoptotic effects of glucocorticoids along with their anti-inflammatory effects should be considered when glucocorticoid eye drops are used in patients with ocular surface disease.

## Introduction

Glucocorticoids (GC) are the most commonly used anti-inflammatory and immunosuppressive drugs, since Philip Hench successfully treated the symptoms of rheumatoid arthritis with cortisone in the late 1940s^1^. One of the immunomodulating mechanisms of GC is the induction of apoptosis in immune cells including T cells^2, 3^ and monocytes/macrophages^4^, a process known as GC-induced apoptosis^5–7^. In addition to immune cells, GC trigger apoptosis in a variety of cells such as osteoblasts, muscle cells, vascular endothelial cells, pericytes, gastric epithelial cells, or pancreatic β cells^5–7^. Conversely, GC inhibit apoptosis and support survival in some cells such as hepatocytes and adipocytes^7–9^. Hence, GC have tissue-specific actions on apoptosis, playing the pro- or anti-apoptotic effects depending on the cell types^7^.

In ophthalmology, GC are widely used via topical administration for the treatment and prevention of various ocular inflammatory and angiogenic diseases^10^. The corneal epithelium is the outermost layer of the eye composed of multiple layers of corneal epithelial cells (CECs), and therefore, CECs are directly exposed to GC applied topically. There are some clinical observations of delayed epithelialization in patients with photorefractive keratectomy or bacterial corneal ulcers after treatment with topical GC^11, 12^. However, there are few studies that directly evaluated the effects of GCs on CECs. In the present study, we investigated the effects of prednisolone, a synthetic glucocorticoid analogue frequently used in the clinic, on the survival, proliferation, apoptosis, and gene expression of CECs.

## Results

### Prednisolone reduces proliferation and promotes apoptosis in CECs

Human CECs (hCECs) were primarily cultured from the corneolimbal rim of donor corneas and treated with prednisolone at various concentrations (0.01 to 100 μg/ml) for 3 days. The culture medium containing the same concentration of prednisolone was exchanged every day. The cells without treatment or with solvent treatment served as negative controls.

**Figure 1:**
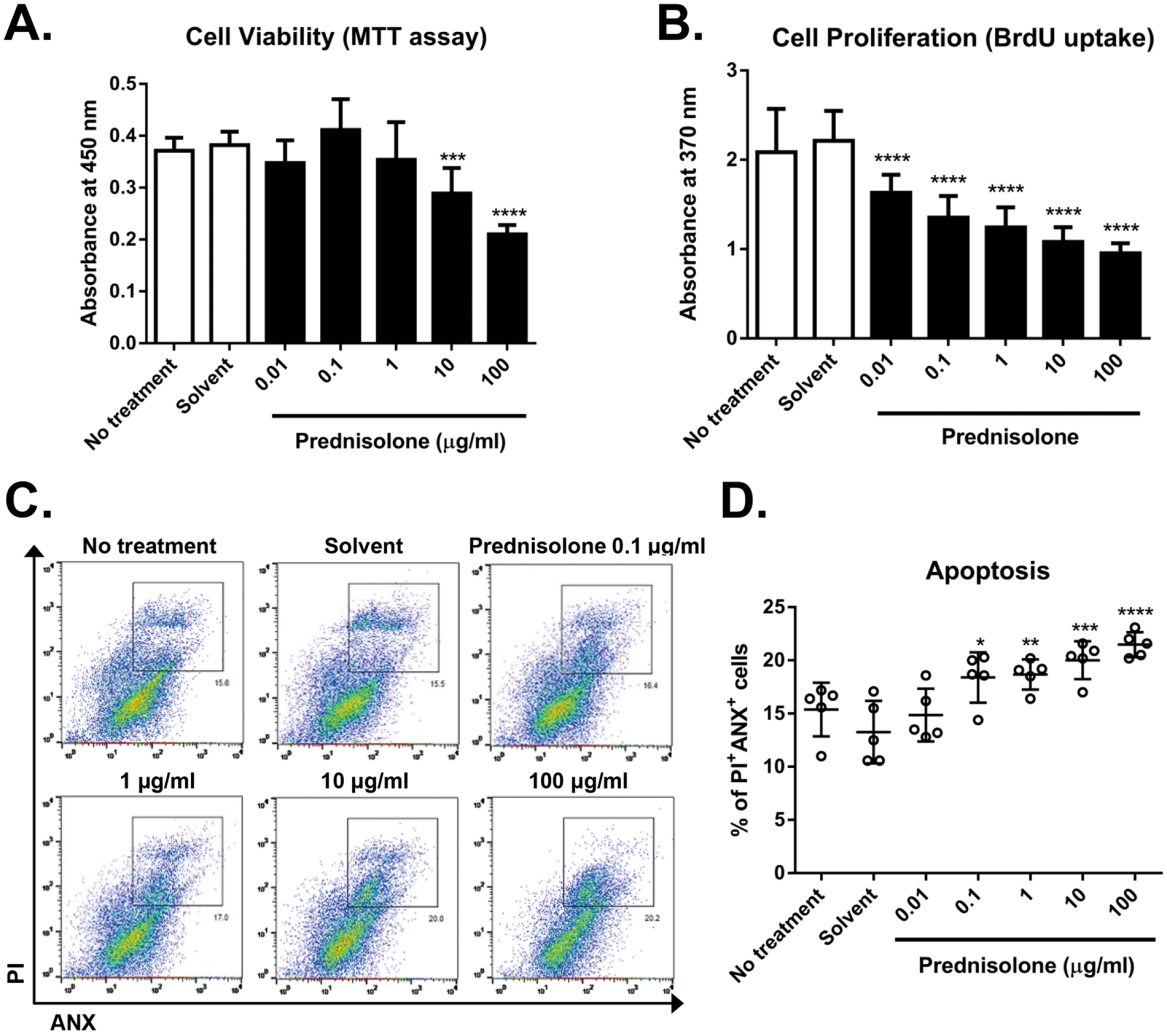
Effects of prednisolone on metabolic activity, proliferation, and apoptosis in human corneal epithelial cells (hCECs). hCECs were cultured in the presence of various concentrations of prednisolone for 3 days and subjected to assays. The cells without any treatment or with solvent treatment alone served as controls. (**A**) MTT assay to measure cell metabolic activity. (**B**) BrdU uptake assay to measure cell proliferation. (**C**) Representative flow cytometry plots for Annexin V (ANX)^+^ and propidium iodide (PI)^+^ cells, indicative of apoptotic cells. (**D**) Quantitative flow cytometry results for ANX^+^PI^+^ cells. Data are presented as mean ±/+ SD from five independent sets of experiments including at least five samples per a set. *p < 0.05, **p < 0.01, ***p < 0.001, ****p < 0.0001.

The metabolic activity of the cells as measured by MTT (3-(4,5-dimethylthiazol-2-yl)-2,5-diphenyl tetrazolium bromide) assay was significantly decreased in hCECs treated with prednisolone (10 and 100 μg/ml), compared to untreated or solvent-treated controls (Fig. 1A). Similarly, the cell proliferation was reduced by prednisolone in a concentration-dependent manner as evaluated by bromodeoxyuridine (BrdU) uptake assay (Fig. 1B). The number of Annexin V (ANX)^+^/propidium iodide (PI)^+^ cells was significantly higher in hCECs treated with prednisolone, indicating the induction of apoptosis (Fig. 1C,D).

### The effects of prednisolone are mediated through the glucocorticoid receptor

**Figure 2:**
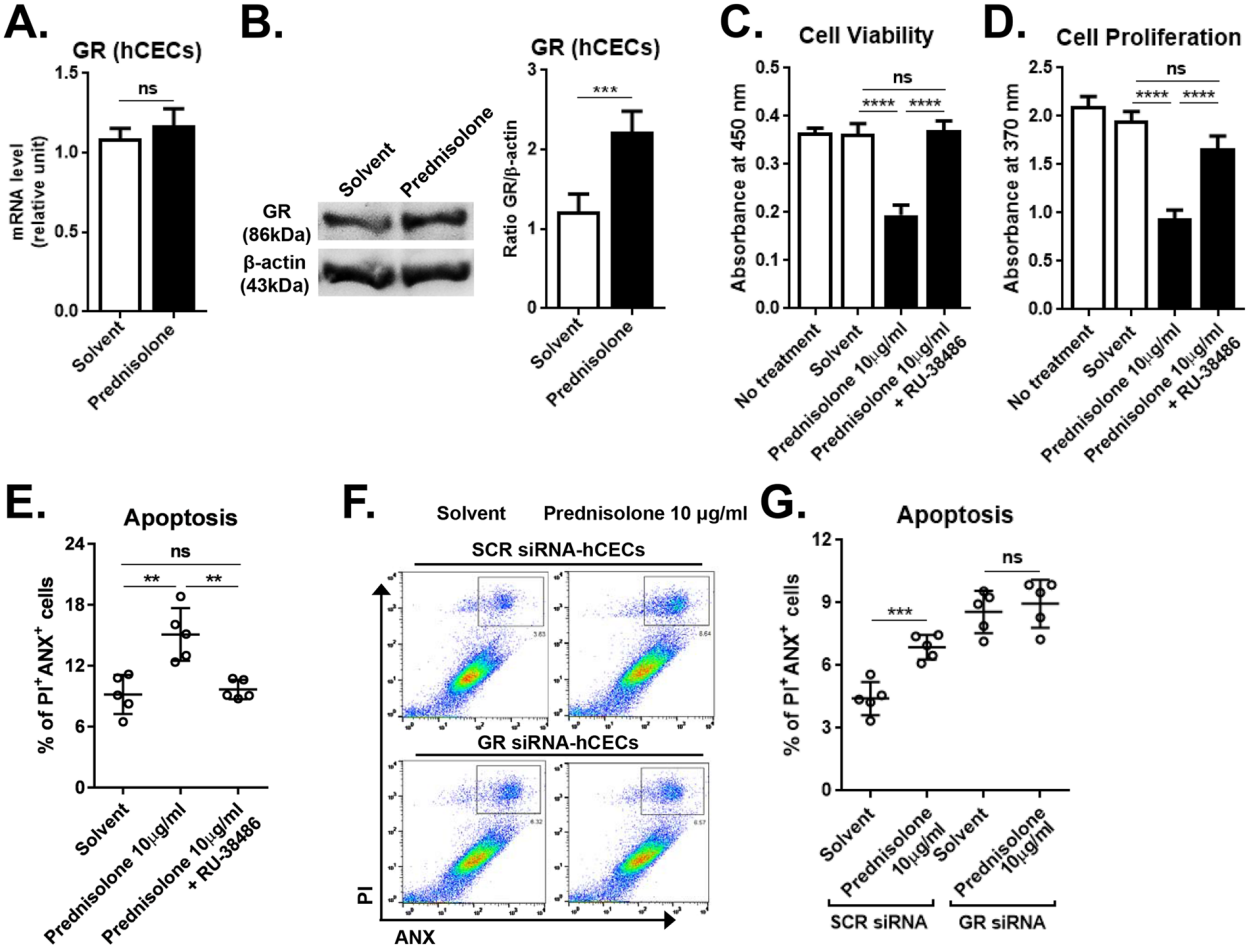
Glucocorticoid receptor (GR) mediates the effects of prednisolone. hCECs were cultured with or without 10 μg/ml prednisolone for 3 days and subjected to assays. (**A**) Real-time RT PCR assay for GR expression. (**B**) Representative image and densitometry analysis of western blot for GR relative to β-actin. (**C**,**D**) MTT assay and BrdU uptake assay to measure cell metabolic activity and proliferation, respectively. (**E**–**G**) Flow cytometric analysis for ANX^+^PI^+^ cells to measure cell apoptosis. The GR antagonist RU-38486 was added to some cultures (**E**). The percentage of ANX^+^PI^+^ cells was measured in hCECs with GR siRNA or scrambled (SCR) siRNA transfection (**F**,**G**). Data are presented as mean ±/+ SD from five independent sets of experiments including at least five samples per a set. **p < 0.01, ***p < 0.001, ****p < 0.0001, ns: not significant.

GC transduce their actions by binding to the glucocorticoid receptor (GR)^13^. The GR isoforms are almost ubiquitously expressed in cells and tissues, and it was previously shown that hCECs express GR^14^. Consistent with these observations, hCECs which we primarily cultured from the corneoscleral limbus expressed GR at both mRNA and protein levels as assessed by real-time RT PCR and western blot (Fig. 2A,B). Interestingly, the treatment with prednisolone 10 μg/ml further increased the level of GR protein in hCECs, compared to the cells treated with solvent alone (Fig. 2B). Therefore, we next determined whether prednisolone affects the metabolic activity, proliferation, and apoptosis in hCECs through GR. Results revealed that prednisolone did not reduce the metabolic activity and proliferation in hCECs treated with GR antagonist RU-38486 (Fig. 2C,D). Similarly, the effects of prednisolone on apoptosis induction in hCECs were blocked either by RU-38486 (Fig. 2E) or in the cells with GR siRNA knockdown (Fig. 2F,G), indicating that GR is required for the pro-apoptotic effects of prednisolone on hCECs.

### Prednisolone increases mitochondrial reactive oxygen species (mtROS)

**Figure 3:**
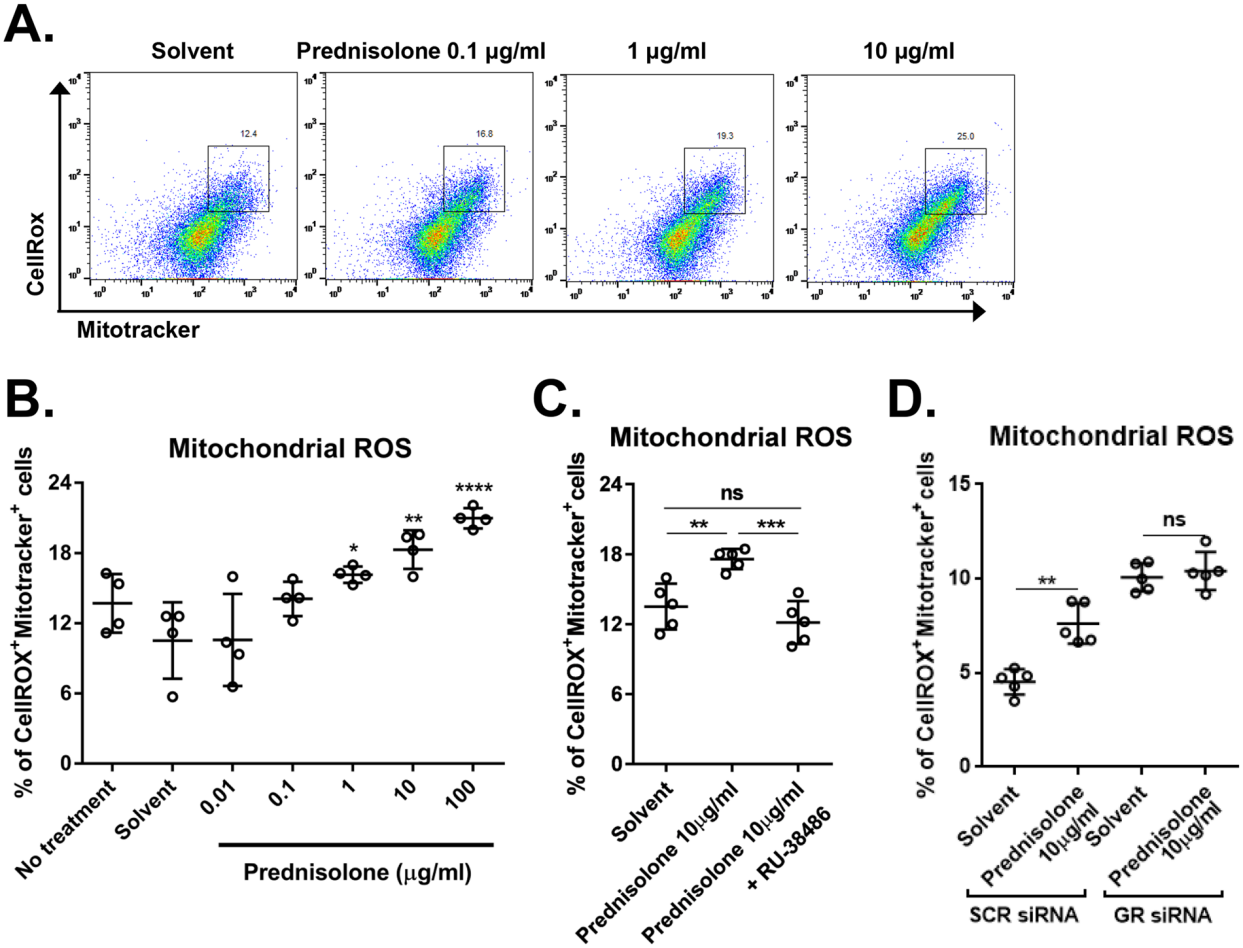
Effects of prednisolone on mitochondrial reactive oxygen species (ROS). (**A**) Representative flow cytometry plots for hCECs after staining with CellROX^TM^ Deep Red and MitoTracker Green dyes. (**B**–**D**) Quantitative flow cytometry results for CellROX^+^MitoTracker^+^ cells as a measure of mitochondrial ROS in hCECs treated with prednisolone. The glucocorticoid receptor (GR) antagonist RU-38486 was added to some cultures (**C**). The mitochondrial ROS was also measured in hCECs transfected with GR siRNA or control SCR siRNA (**D**). Data are presented as mean ± SD from at least four independent experiments. *p < 0.05, **p < 0.01, ***p < 0.001, ****p < 0.0001, ns: not significant.

GC induce apoptosis via several mechanisms in the cell- and tissue-type specific manners^5, 6^. To investigate the mechanism underlying GC-induced apoptosis in hCECs, we examined the level of mitochondrial reactive oxygen species (mtROS) in hCECs treated with prednisolone. The mtROS level was determined by flow cytometry after staining the cells with CellROX dye that fluoresces upon oxidation by ROS and MitoTracker Green dye that stains total mitochondria regardless of mitochondrial membrane potential. Results showed that the percentage of CellROX^+^MitoTracker^+^ cells was markedly higher in hCECs treated with prednisolone than in untreated cells or the cells treated with solvent alone, suggesting an increase in mtROS (Fig. 3A,B). The effects of prednisolone on mtROS levels were concentration-dependent (Fig. 3A,B), and significantly abrogated by an addition of RU-38486 to the culture (Fig. 3C) or in the cells with siRNA knockdown of GR (Fig. 3D).

### mtROS is involved in the prednisolone-induced apoptosis

**Figure 4:**
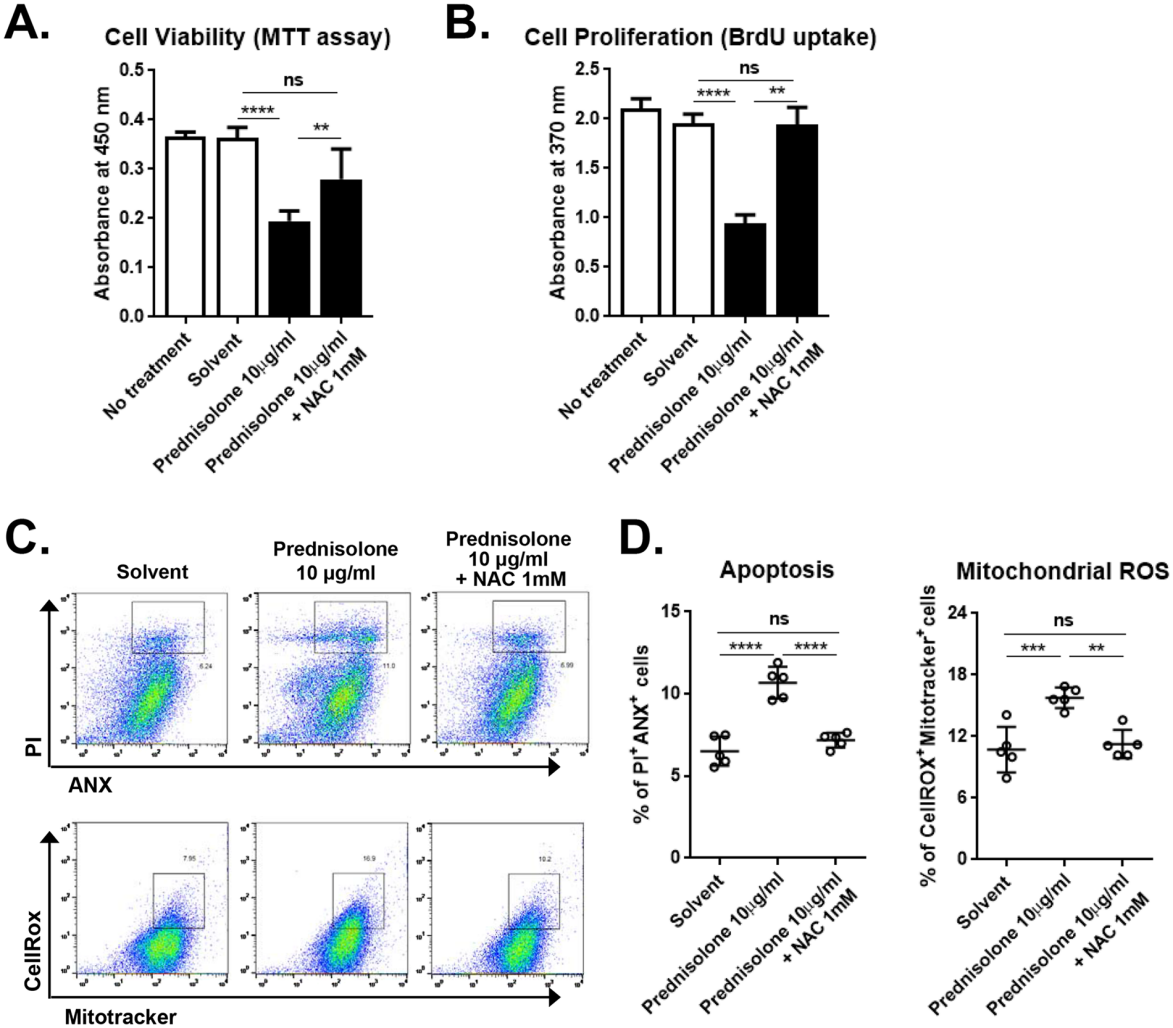
The mitochondrial ROS mediates the effects of prednisolone. (**A**,**B**) MTT and BrdU uptake assays in hCECs treated with 10 μg/ml prednisolone in the presence or absence of N-acetylcysteine (NAC). (**C**,**D**) Representative and quantitative flow cytometry results for ANX^+^PI^+^ cells and CellROX^+^MitoTracker^+^ cells. Data are presented as mean ± SD from five independent experiments. **p < 0.01, ***p < 0.001, ****p < 0.0001, ns: not significant.

We next investigated whether the increase of mtROS might be involved in detrimental effects of prednisolone on the survival and proliferation of hCECs. The treatment of hCECs with N-acetylcysteine (NAC) negated the effects of prednisolone in suppressing the metabolic activity and proliferation of hCECs (Fig. 4A,B). Also, the increases in apoptosis and mtROS levels in the prednisolone-treated hCECs were markedly abrogated by NAC treatment (Fig. 4C,D), suggesting that the oxidative stress mediates the apoptosis induction in hCECs by prednisolone.

### Prednisolone induces active caspase-3 and -9, but not -8

**Figure 5:**
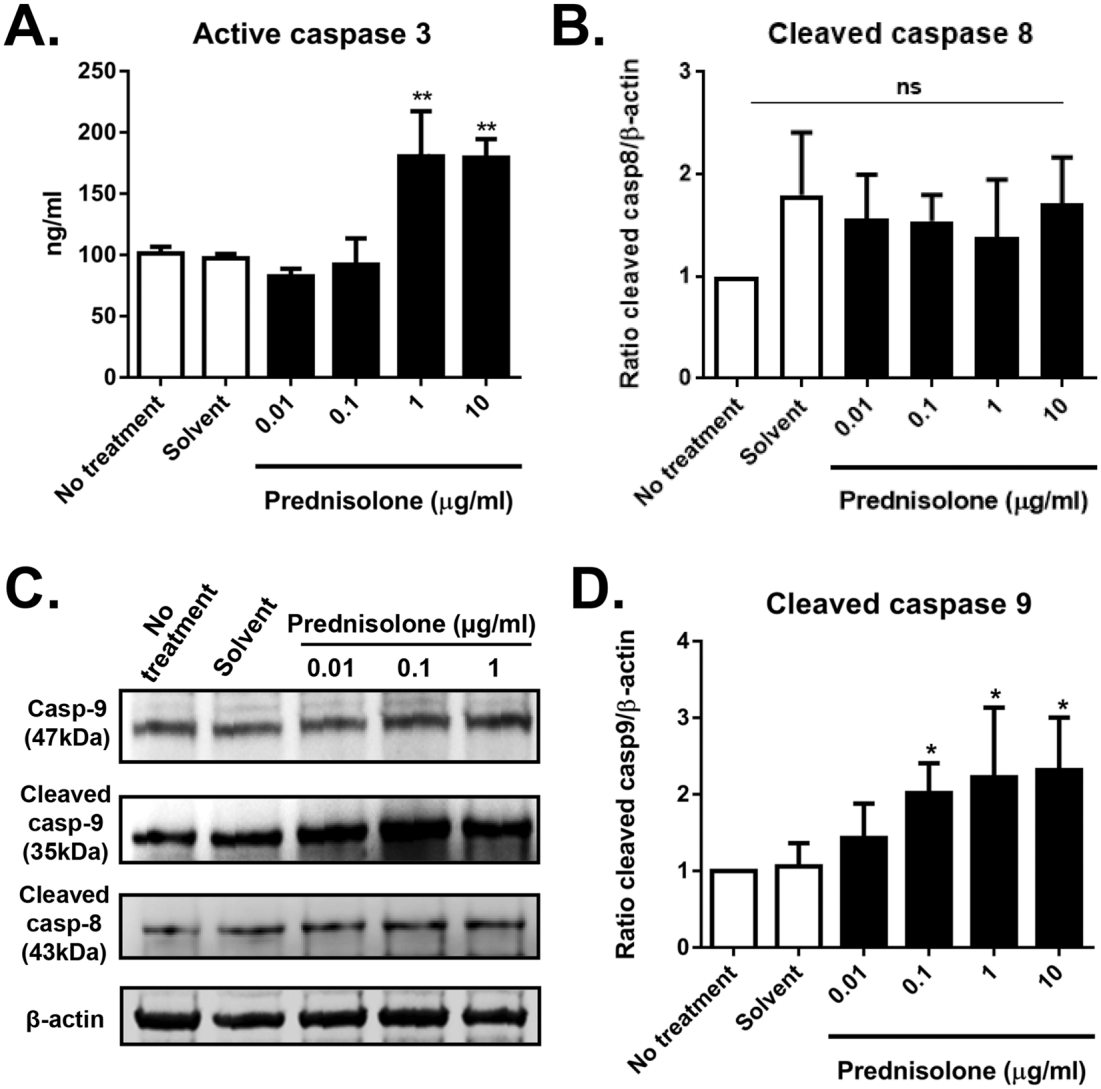
Effects of prednisolone on caspase activity and cleavage. (**A**) ELISA results for caspase-3 activity. (**B**) Densitometric analysis of the ratio of cleaved casepse-8 relative to β-actin. (**C**) Representative images of western blot analysis for pro-caspase-9 (Casp-9), cleaved caspase-9, cleaved caspase-8, and β-actin. Full, uncropped gel images are shown. (**D**) Densitometric analysis of the ratio of cleaved caspase-9 relative to β-actin. Data are presented as mean + SD from six independent sets of experiments. *p < 0.05, **p < 0.01, ns: not significant.

Next, we evaluated the activity and proteolytic processing of caspases because activation of caspases plays a role in apoptosis^15^. Prednisolone treatment significantly increased the activity of caspase-3, an effector caspase in apoptosis pathway (Fig. 5A). Further assays for the initiator caspases-8 and -9 using western blotting showed that the protein level of cleaved caspase-9 was markedly increased by prednisolone in a concentration-dependent manner, while the expression of cleaved caspase-8 was not changed by prednisolone (Fig. 5B–D).

### Prednisolone represses pro-inflammatory cytokine transcription

**Figure 6:**
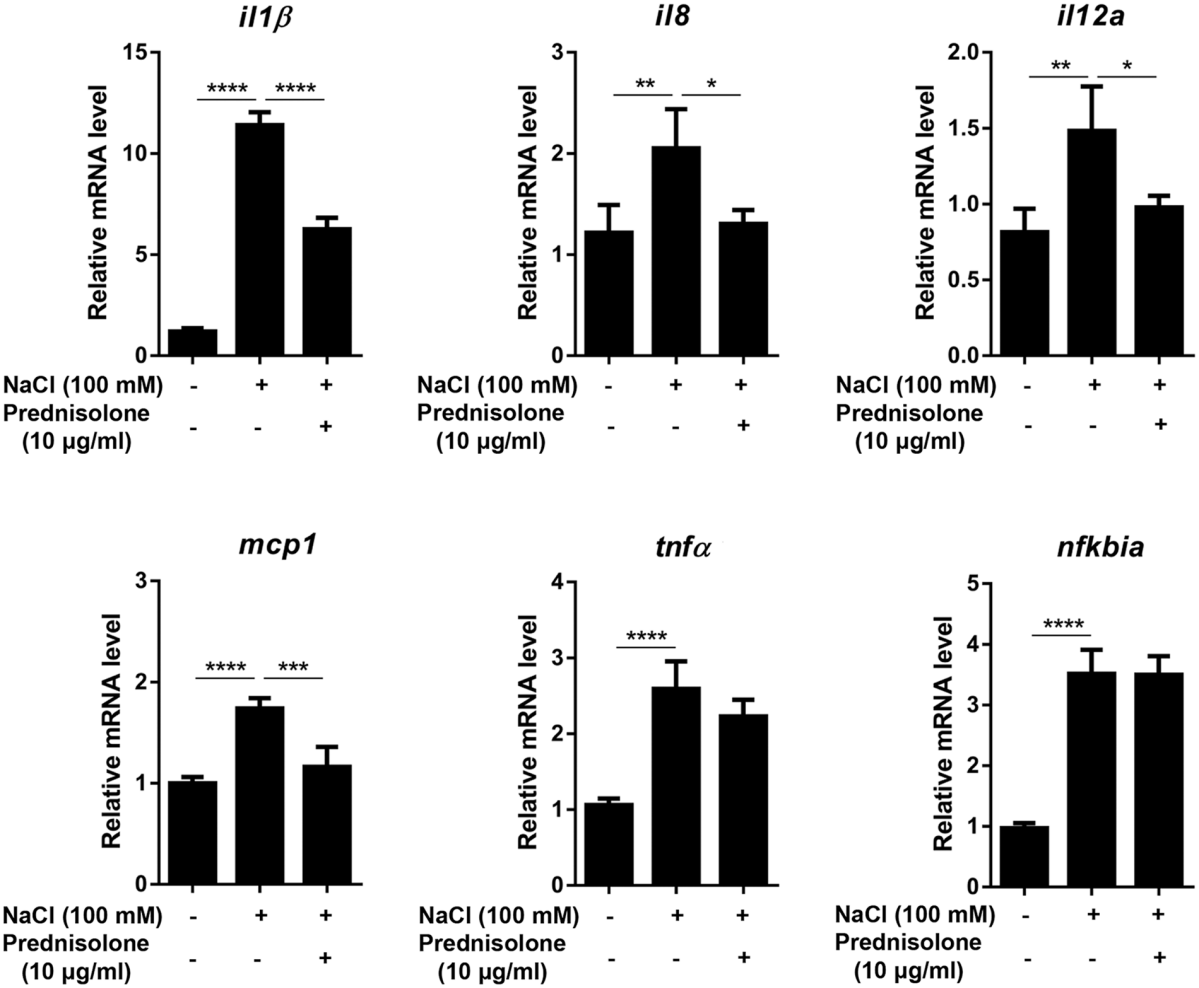
Effect of prednisolone on inflammatory cytokine expression in hCECs stimulated by hyperosmolarity. hCECs were stimulated by 100 mM NaCl in the presence or absence of prednisolone (10 µg/ml) for 3 days, and the mRNA levels of IL-1β, IL-8, IL-12A, MCP-1, TNF-α, and NFKBIA were measured by real-time RT PCR. Data are presented as mean + SD from five independent experiments. *p < 0.05, **p < 0.01, ***p < 0.001, ****p < 0.0001.

GC suppress transcription of inflammatory genes through a process termed “transrepression”, leading to the anti-inflammatory and immunosuppressive effects^16, 17^. Therefore, we examined whether prednisolone affects the transcription profiles of pro-inflammatory and cell-specific genes in hCECs. Real-time RT PCR demonstrated that prednisolone did not change the mRNA levels of inflammation-related cytokines (TNF-α, IL-1β, IL-6, IL-12A, and TGF-β1) or epithelial stem cell markers (P63 and ABCG2) in hCECs at the steady-state (Supplementary Fig. 1). Next, hCECs were subjected to hyperosmolar stress by culturing the cells in hyperosmolar media (100 mM NaCl). Hyperosmolarity causes damage to corneal epithelial cells in various disease conditions such as dry eye disease or diabetes^18–20^. Upon hyperosmotic stimulation, the mRNA levels of pro-inflammatory cytokines, IL-1β, IL-8, IL-12A, MCP-1, TNF-α, and NFKBIA, were markedly increased in hCECs. Prednisolone treatment significantly reduced the levels of IL-1β, IL-8, IL-12A, and MCP-1 transcripts in hCECs stimulated with hyperosmolarity (Fig. 6). However, the transcript levels of epithelial stem cell markers (P62 and ABCG2) were not altered by prednisolone (Supplementary Fig. 2).

### Topical prednisolone increases apoptosis in corneal epithelium in dry eye mice

**Figure 7:**
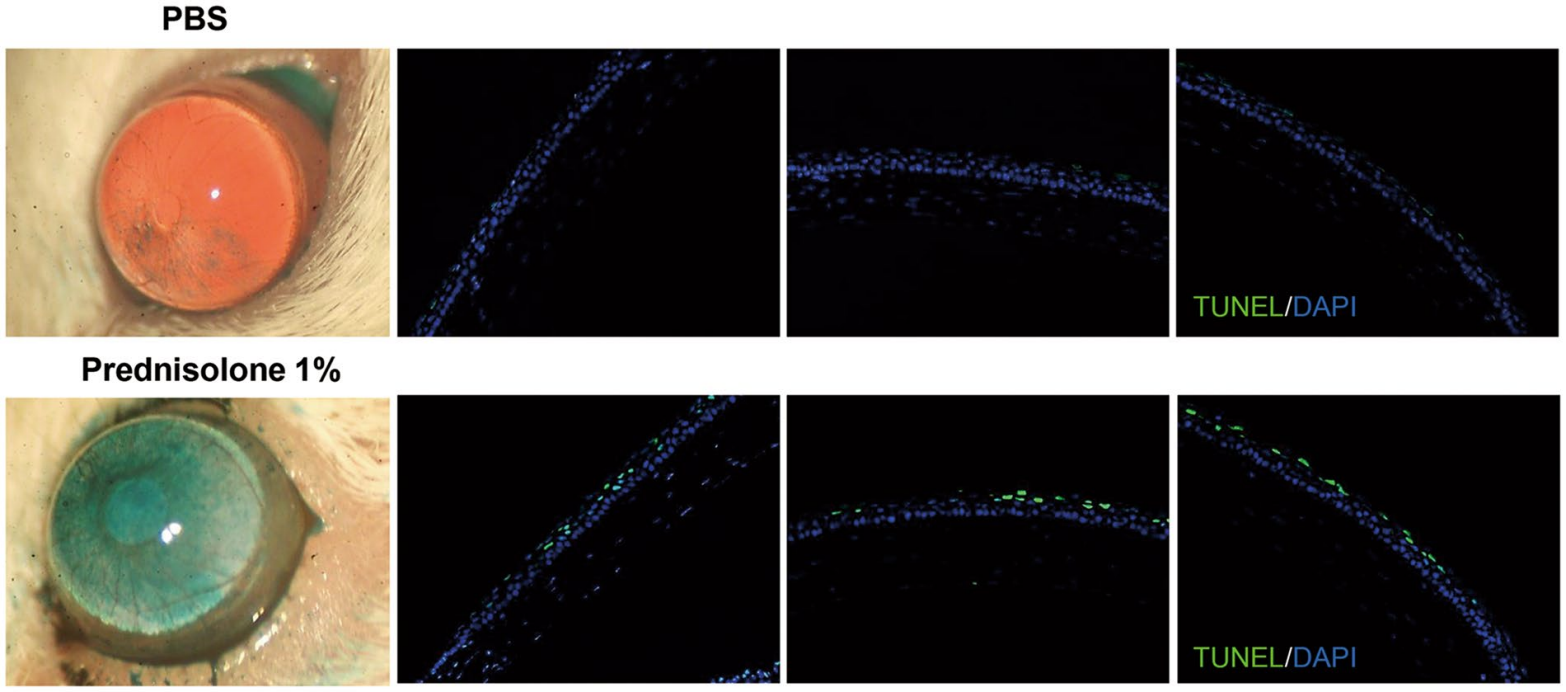
Effect of 1% prednisolone eye drops on the corneal epithelium in dry eye mice. Either 1% prednisolone acetate (Pred forte^®^, Allergan, Irvine, TX) or the same volume of phosphate buffered solution (PBS) was topically applied QID for 7 days to eyes of 12-week-old female NOD.B10.H2^b^ mice (six animals in each group). Representative corneal photographs after lissamine green staining and TUNEL staining of the corneal epithelium are shown. Original magnification x100.

To further confirm the pro-apoptotic effects of prednisolone on the corneal epithelium *in vivo*, prednisolone 1% eye drops were topically applied QID in NOD.B10.H2^b^ mice, a murine model of human ocular Sjögren’s syndrome and dry eye disease^21^. Seven days later, the corneas were examined for epithelial defects by lissamine green staining and for apoptosis by TUNEL (terminal deoxynucleotidyl transferase dUTP nick end labeling) staining. Results revealed that prednisolone treatment markedly increased punctate epithelial erosions and TUNEL^+^ apoptotic cells in the corneal epithelium of the mice (Fig. 7).

## Discussion

Our results demonstrate that prednisolone reduces the survival of hCECs by suppressing proliferation and inducing apoptosis. The prednisolone-induced apoptosis of hCECs occurred through GR and the intrinsic pathway involving mtROS, caspase-9 and -3.

Apoptosis is the process of programmed cell death and characterized by distinct morphological characteristics and energy-dependent biochemical mechanisms^22^. The mechanisms of apoptosis, albeit highly complex, include two main pathways: the extrinsic or death receptor pathway and the intrinsic or mitochondrial pathway^7, 22^. The extrinsic pathway is initiated by transmembrane receptor-mediated interactions, resulting in the auto-catalytic activation of procaspase-8. The intrinsic pathway is initiated by non-receptor-mediated stimuli that cause changes in the inner mitochondrial membrane leading to caspase-9 activation. Both extrinsic and intrinsic pathways converge on the execution pathway which is mediated by the cleavage of caspase-3, DNA fragmentation, formation of apoptotic bodies, and expression of ligands for phagocytic cell receptors. We here found that prednisolone increased the number of ANX^+^PI^+^ or TUNEL^+^ cells, the level of mtROS, caspase-3 activity, and the cleavage of caspase-9 in hCECs. These effects of prednisolone on apoptosis induction were dependent on GR expression and reversed by mtROS inhibition with NAC. Therefore, our findings are consistent with previous studies in that GC are potent inducers of cell apoptosis and GC-induced apoptosis is mediated by activation of GR and the intrinsic apoptotic pathway^7, 22, 23^.

Several previous studies investigated the effects of GC on the cornea. Topical application of GC to the cornea *in vivo* has been shown to delay the epithelial wound healing in rabbits, cats, and humans^11, 12, 24, 25^. Also, *in vitro* studies demonstrated that cultured corneal epithelial, endothelial cells, and keratocytes express GR, and dexamethasone induces apoptosis in cultured corneal cells^14, 26, 27^. A study by Bourcier *et al*.^14^ showed that dexamethasone induced apoptosis in cultured hCECs at both low and high concentrations, while it increased proliferation at low concentrations and suppressed at high concentrations. However, they examined the apoptosis by nucleus labeling of the cells with Hoechst 33258 and counting the cells with fragmented nuclei. Also, they evaluated cell proliferation by MTT assay which is the measure of the metabolic activity of the cells, not proliferation. In the present study, we measured the number of ANX^+^PI^+^ cells, mtROS level, the activity and cleavage of caspases to analyze the apoptosis and its pathway, and examined the amount of BrdU uptake to assess proliferation. We found that prednisolone reduced proliferation in hCECs at concentrations of 0.01 to 100 μg/ml and induced apoptosis at 0.1 to 100 μg/ml. Considering that 1% prednisolone is the most commonly used concentration in the clinical setting, our study indicates that prednisolone may play a negative role in the corneal epithelial cell viability at therapeutic concentrations.

Another notable finding of our study is that prednisolone reduced the transcription of major pro-inflammatory cytokines in hCECs that was increased after hyperosmolar stimulation. This finding is consistent with the anti-inflammatory effects of GC through transcriptional repression of inflammatory cytokines^16, 17^. It has been previously shown that damaged corneal cells can initiate and amplify inflammation by producing inflammatory cytokines that would act as damage-associated molecular patterns (DAMPs)^28^. Hence, it is possible that prednisolone might contribute to the initial suppression of inflammation in the cornea by reducing the levels of inflammatory cytokines and DAMPs produced by CECs in response to injury.

Ocular surface diseases (OSD) are accompanied by corneal epithelial defects and inflammation. GC including prednisolone may impede the corneal epithelial cell integrity while suppressing inflammation as shown in our study. Therefore, the potential pro-apoptotic effects and anti-inflammatory effects of GC on the cornea should be considered when GC are used for the treatment of patients with OSD.

## Methods

The experimental protocol was approved by the Institutional Review Board of Seoul National University Hospital and complied with the tenets of the Declaration of Helsinki for Research Involving Human Tissue.

### Cells and reagents

Primary hCECs were obtained with informed consent and cultured from the corneolimbal rim of donor corneas as we previously reported. Briefly, the tissue was treated with 0.05% trypsin and 0.01% EDTA at 37 °C to separate epithelial cells from the stroma. The cells were then seeded on a 3T3 fibroblast feeder layer (NIH/3T3 cell line, ATCC, Manassas, VA) that had been pre-treated with 4 µg/ml mitomycin C (Sigma-Aldrich, St Louis, MO) for 2 h, and cultured in supplemented hormonal epithelial media (SHEM) containing DMEM/F12 (Lonza, Valais, Switzerland), 10% fetal bovine serum (Welgene, Daegu, Korea), 0.5% penicillin-streptomycin, 5 µg/ml insulin, 30 ng/ml cholera toxin, 10 ng/ml epidermal growth factor, and, 0.18 mM adenine, 4 mM glutamine, and 2 nM triiodothyronine. Passage 2 cells were used for experiments.

Prednisolone (Cat no. P6004, Sigma-Aldrich) was dissolved at a concentration of 50 mg/ml in solvent (CHCl_3_: MeOH = 1:1), and added to the culture media at the concentrations of 0.01, 0.1, 1, 10, and 100 µg/ml. Control groups of hCECs were cultured in the media without any treatment or in the media containing the solvent alone. In all experiments, the solvent concentration in the culture media was maintained at <0.2%. The media containing the same concentration of prednisolone or solvent was exchanged every day.

For the GR antagonist treatment, RU-38486 (mifepristone) was purchased from Sigma-Aldrich (M8046) and added to the culture at the concentration of 10^−5^ M.

For GR knockdown, hCECs were transfected with siRNA for GR (Cat no. sc-35505, Santa Cruz Biotechnology, Santa Cruz, CA) or scrambled siRNA (Cat no. sc-37007, Santa Cruz Biotechnology) with a commercial kit (Lipofectamine RNAiMAX reagent, Thermo Fisher Scientific, Grand Island, NY) and cultured in serum-free Opti-MEM (Thermo Fisher Scientific) for 4 h at 37 °C. To confirm the successful knock-down of GR expression, RNA was extracted from the cells at 24 h after the start of transfection (RNeasy Mini kit, Qiagen, Valencia, CA) and assayed for GR by real-time RT-PCR. The knockdown efficiency of GR in hCECs was 80.2%. At the same time-point, GR siRNA or scrambled siRNA-transfected hCECs were treated with prednisolone or solvent.

For NAC treatment, *N*-Acetyl-L-cysteine was obtained from Sigma-Aldrich and added to the culture at 1 mM.

For hyperosmolar stimulation, 100 mM NaCl was added to the culture to produce 450 mOsM.

### MTT assay, BrdU uptake and caspase-3 activity measurement

The hCECs (5 × 10^4^ cell per well) were seeded and cultured in SHEM in a 96-well plate for 24 h. Prednisolone or solvent was added to the culture, and the cells were cultured for 3 days with the media exchanged every day. The metabolic activity of the cells was measured using MTT assay (Cell Counting Kit-8, Dojindo Laboratories, Kumamoto, Japan). The cell proliferation was quantitated by measuring BrdU incorporation in cells using colorimetric immunoassay (Cell Proliferation ELISA, BrdU, Roche, Indianapolis, IN) as per as the manufacturer’s protocol. The activity of caspase-3 was measured using Caspase-3 Active Human ELISA Kit (Thermo Fisher Scientific).

### Apoptosis assay and ROS measurement

The hCECs (1 × 10^5^ cell per well) were seeded and cultured in SHEM in a 6-well plate for 24 h. The cells were then treated with prednisolone or solvent and further cultured for 3 days. For apoptosis assay, the cells were stained with a combination of PI and Annexin V (Cat no. 556547, FITC Annexin V Apoptosis Detection Kit I, BD Pharmingen™, San Diego, CA) at room temperature for 5 min and analyzed by flow cytometry (S1000EXi Flow Cytometer, Stratedigm, Inc., San Jose, CA). For mitochondrial ROS measurements, the cells were stained with both CellROX dye (5 μM, CellROX^TM^ Deep Red Reagent, Invitrogen) and MitoTracker Green dye (100 nM, MitoTracker Green FM Dye, Invitrogen) at 37 °C for 30 min, and analyzed for fluorescence using flow cytometry. The data obtained from flow cytometer were analyzed using Flowjo software (Tree Star, Ashland, OR).

### Western blotting

For protein extraction, the cells were harvested after 24 h of prednisolone treatment and sonicated on ice in PRO-PREP™ Protein Extraction Solution (Cat No. 17081, iNtRON BIOTECHNOLOGY, Inc, Korea). A total of 30 µg protein per sample was fractionated by Tris-Glycine-PAG, SDS Precast gel, 8~16% (Cat No. KG75505, Komabiotech, Seoul, Korea), transferred to Immobilon-P membrane, PVDF (Cat No. IPVH00010, Millipore, Corporation Canada), and then blotted with antibodies against GR (ab55189, Abcam, Cambridge, UK), cleaved caspase-8 (Asp391, 18C8, Cell Signaling Technology, Danvers, MA), caspase-9 (Cell Signaling Technology), and β–actin (sc-130656, Santa Cruz Biotechnology, Santa Cruz, CA).

### Real-time RT PCR

For RNA extraction, the cells were lysed in RNA isolation reagent (RNA Bee, Tel-Test Inc., Friendswood, TX), and total RNA was then extracted using RNeasy Mini kit (Qiagen, Valencia, CA). First-strand cDNA was synthesized by reverse transcription (High Capacity RNA-to-cDNA Kit, Applied Biosystems, Carlsbad, CA). Real-time amplification was performed using TaqMan^®^ Universal PCR Master Mix (Applied Biosystems) in an automated instrument (ABI 7500 Real Time PCR System, Applied Biosystems) for the following molecules: GR, TNF-α, IL-1β, IL-6, IL-8, IL-12A, MCP-1, NFKBIA, TGF-β1, P62, and ABCG2. Values were normalized to 18s rRNA and expressed as fold changes relative to controls. Human PCR probe sets were commercially purchased (TaqMan^®^ Gene Expression Assay Kits, Applied Biosystems).

### Animal experiments

Animal experiment was approved by the Institutional Animal Care and Use Committee of Seoul National University Hospital Biomedical Research Institute (IACUC No. 13-0162). Animals were treated in strict accordance with the ARVO statement for the use of animals in ophthalmic and vision research.

Twelve-week-old female NOD.B10.H2^b^/J mice (Jackson Laboratories, Bar Harbor, ME) were treated with topical administration of 1% prednisolone acetate (Pred forte^®^, Allergan, Irvine, CA) or PBS (phosphate buffered solution) QID for 7 days. The corneal epithelial defects were observed and photographed after lissamine green staining (3% Lissamine™ Green B, Sigma-Aldrich). The cornea was then excised and fixed in formalin. The tissue was sliced into 4-μm-thick sections and subjected to TUNEL staining (ApopTag^®^ Red *in situ* Apoptosis Detection Kit, EMD Millipore, Billerica, MA). A DAPI (5ug/ml, Bisbenzimide H 33342, Sigma-Aldrich) was used for nuclear counter staining.

### Statistical analysis

Experiments were independently performed at least five times. GraphPad Prism^®^ Software (La Jolla, CA) was used for statistical tests. Data were analyzed by Student’s *t* test to compare means of two groups or by one-way ANOVA to compare means of three or more groups. Tuckey’s Honestly Significant Difference (HSD) test was used for a follow-up pairwise comparison. The data are presented as the mean +/± SD. Differences were considered significant at *p* < 0.05.

## Electronic supplementary material

Supplementary Information
