# Supplementary material for: Prednisolone induces apoptosis in corneal epithelial cells through the intrinsic pathway
Source: Sci Rep. 2017 Jun 23;7:4135. doi: 10.1038/s41598-017-04509-8 (PMC5482844; doi:10.1038/s41598-017-04509-8)

Prednisolone induces apoptosis in corneal epithelial cells through the intrinsic pathway.

Jin Suk Ryu,[1] Jung Hwa Ko,[1] Mee Kum Kim,[1, 2] Won Ryang Wee,[1, 2] and Joo Youn Oh[1, 2, *]

[1]Laboratory of Ocular Regenerative Medicine and Immunology, Biomedical Research Institute, Seoul National University Hospital, 101 Daehak-ro, Jongno-gu, Seoul 110-744, Korea

[2]Department of Ophthalmology, Seoul National University Hospital, 101 Daehak-ro, Jongno-gu, Seoul 110-744, Korea

* jooyounoh77@gmail.com

***Correspondence to:** Joo Youn Oh, MD, PhD. Department of Ophthalmology, Seoul National University Hospital, 101 Daehak-ro, Jongno-gu, Seoul, 110-744, Korea. Tel: 82-2-2072-0836; Fax: 82-2-741-3187; E-mail: jooyounoh77@gmail.com

Supplementary Figure 1. Effect of prednisolone on inflammatory cytokine expression in hCECs at steady state. Real-time RT PCR analysis for inflammatory cytokines (TNF-α, IL-1β, IL-6, IL-12A, and TGF-β1) and epithelial stem cell-specific markers (P63 and ABCG2) in hCECs treated with various concentrations of prednisolone. Data are presented as mean + SD from five independent experiments.

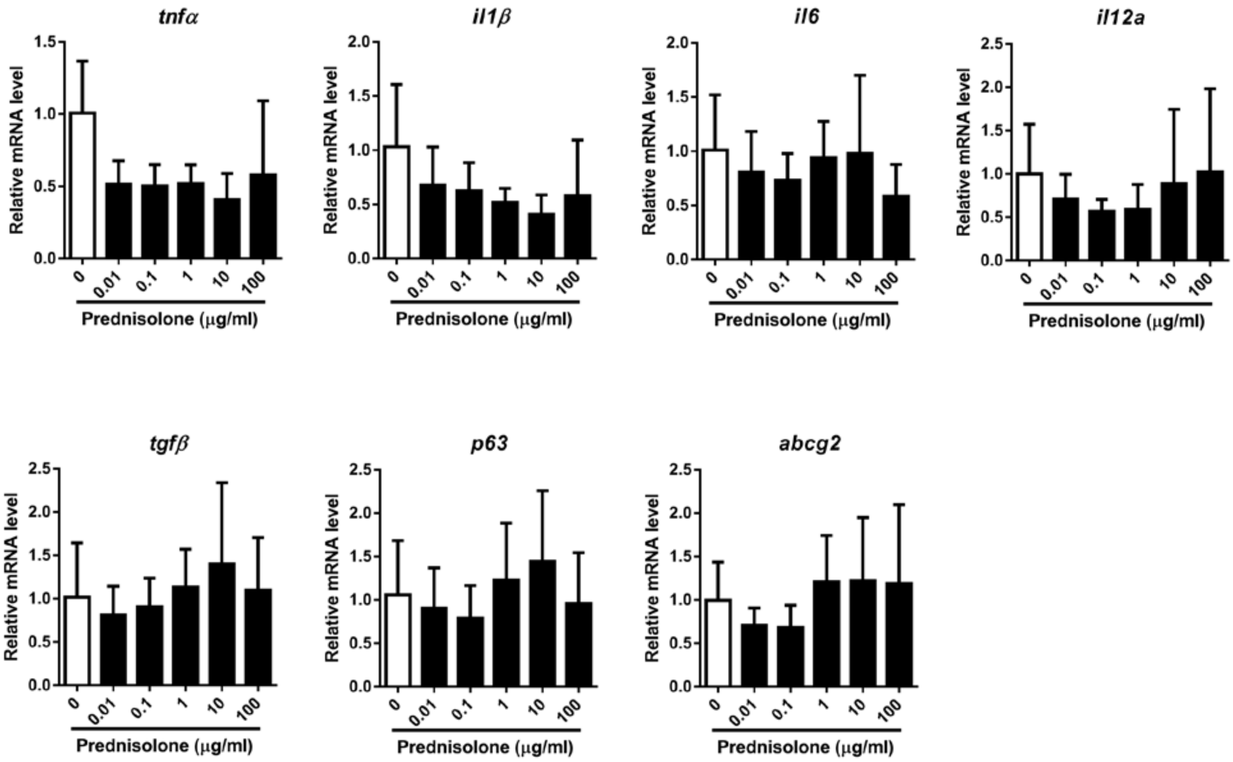

Supplementary Figure 2. Effect of prednisolone on epithelial stem cell-specific markers in hCECs stimulated with hyperosmolarity. Real-time RT PCR analysis for epithelial stem cell-specific markers (P63 and ABCG2) in hCECs treated by 100 mM NaCl in the presence or absence of prednisolone (10 µg/ml) for 3 days. Data are presented as mean + SD from five independent experiments. ** $p < 0.01$.

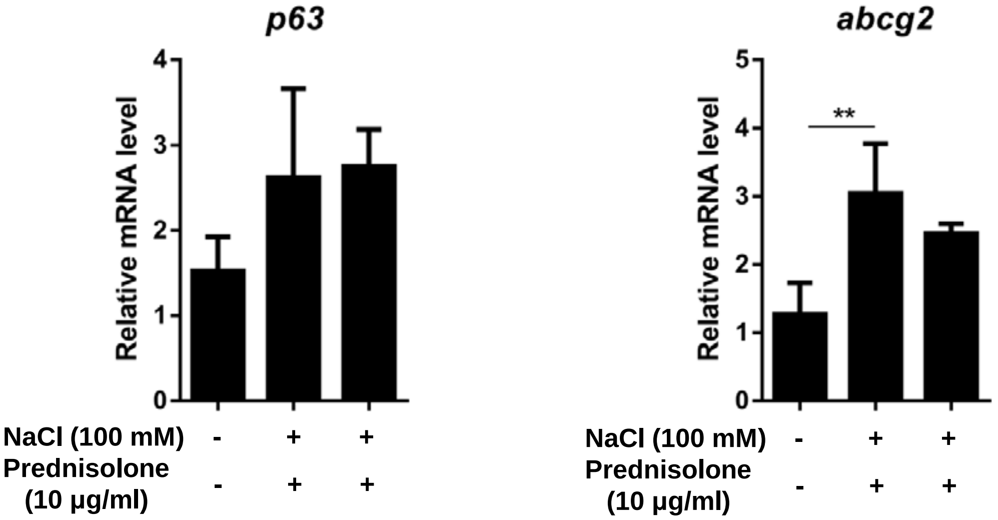

Supplement: Supplementary file 1 — Supplementary Information [file 41598_2017_4509_MOESM1_ESM.pdf]
